# Voriconazole-Loaded Nanohydrogels Towards Optimized Antifungal Therapy for Cystic Fibrosis Patients

**DOI:** 10.3390/pharmaceutics17060725

**Published:** 2025-05-30

**Authors:** Shaul D. Cemal, María F. Ladetto, Katherine Hermida Alava, Gila Kazimirsky, Marcela Cucher, Romina J. Glisoni, María L. Cuestas, Gerardo Byk

**Affiliations:** 1 Department of Chemistry, Bar-Ilan University, Ramat Gan 5290002, Israel; shaulcemal11@gmail.com (S.D.C.); gilakazimirsky@gmail.com (G.K.); 2 Instituto de Investigaciones en Microbiología y Parasitología Médica (IMPaM), CONICET, Universidad de Buenos Aires, Buenos Aires 1121, Argentina; mflorencialadetto@gmail.com (M.F.L.); katherinehermidaalava@gmail.com (K.H.A.); marcecucher@gmail.com (M.C.); 3 Centro de Investigación y Desarrollo en Fermentaciones Industriales (CINDEFI), Laboratorio de Nanobiomateriales, Departamento de Química, Facultad de Ciencias Exactas, Universidad Nacional de La Plata (UNLP)-CONICET (CCT La Plata), Buenos Aires 1121, Argentina; 4 Instituto de Nanobiotecnología (NANOBIOTEC), CONICET, Universidad de Buenos Aires, Buenos Aires 1121, Argentina; romy.glisoni@gmail.com

**Keywords:** voriconazole, biodegradable nanohydrogels, cystic fibrosis, CF mucus model, drug delivery, drug release

## Abstract

**Background/Objectives**: Filamentous fungi, in particular the species *Aspergillus*, *Scedosporium*, and *Exophiala*, frequently colonize the lungs of cystic fibrosis (CF) patients. Chronic colonization is linked to hypersensitivity reactions and persistent infections leading to a significant long-term decline in lung function. Azole antifungal therapy such as voriconazole (VRC) slows disease progression, particularly in patients with advanced CF; however, excessive mucus production in CF lungs poses a diffusional barrier to effective treatment. **Methods**: Here, biodegradable nanohydrogels (NHGs) recently developed as nanocarriers were evaluated for formulating VRC as a platform for treating fungal infections in CF lungs. The NHGs entrapped up to about 30 μg/mg of VRC, and physicochemical properties were investigated via dynamic laser light scattering and nanoparticle tracking analysis. Diameters were 100–400 nm, and excellent colloidal stability was demonstrated in interstitial fluids, indicating potential for pulmonary delivery. Nano-formulations exhibited high in vitro cytocompatibility in A549 and HEK293T cells and were tested for the release of VRC under two different sink conditions. **Results**: Notably, the antifungal activity of VRC-loaded nanohydrogels was up to eight-fold greater than an aqueous suspension drug against different fungal species isolated from CF sputum, regardless of the presence of a CF artificial mucus layer. **Conclusions**: These findings support the development of potent VRC nano-formulations for treating fungal disorders in CF lungs.

## 1. Introduction

Cystic fibrosis (CF) is the most common inherited life-limiting disease among Caucasian people, affecting 90,000 people worldwide [1]. This single-gene disease, with autosomal recessive inheritance is caused by a mutation in the CF gene encoding the transmembrane conductance regulator (CFTR) [2], which forms a chloride channel that is critical to efficient mucus transport. Mutations in CFTR disrupt chloride secretion, sodium reabsorption, and water transport, leading to mucus hyper concentration and decreased mucociliary clearance [3].

The resultant dehydrated form of mucus is characterized by a reduced mesh size (60−300 nm) [4] in comparison to physiological mucus (497−503 nm) [5]. Dehydrated mucus secretions lead to endobronchial infection with a narrow spectrum of distinctive bacteria and fungi and an exaggerated inflammatory response, resulting in the development of severe bronchiectasis rather than fibrosis and, eventually, respiratory failure [6]. Therefore, in mucus-related pathologies such as CF, an overexpression of mucins, accumulation of extracellular DNA, and cellular debris, as well as the persistent presence of bacteria and fungi, confer mucus stasis, leading to a vicious cycle of infection and inflammation that can be chronically sustained [7,8]. This pathological mucus strongly limits the absorption of antibiotics, antifungals, and anti-inflammatory drugs, preventing them from reaching target sites [9,10,11,12,13].

Despite great advancements in disease management in recent decades, pulmonary failure remains the main cause of morbidity and mortality in CF patients [2]. Recurrent lung infections that lead to chronic airway inflammation and respiratory failure remain the major prognostic problem accounting for over 90% of mortality [14]. Clinical research and therapy mostly focus on the role of bacterial species with little attention to the role of fungi in the pathogenesis of lung function decline, despite their frequent isolation in respiratory samples [15].

Filamentous fungi, especially *Aspergillus*, *Scedosporium*, and *Exophiala* species, are frequent lung colonizers in CF patients [16]. Chronic filamentous fungal colonization is particularly observed at a late stage of the disease and is associated with allergic bronchopulmonary mycosis and chronic infections related to a significant decline in lung function that persists over time [16,17,18,19,20,21,22,23]. Chronic antifungal therapy with azole drugs such as voriconazole (VRC) slows down this progression, mainly in patients with advanced disease [18].

However, the use of VRC is often limited by its poor solubility and bioavailability. VRC is a relatively lipophilic molecule with a Log D = 1.8 at pH 7.4, with an epithelial lining fluid/plasma ratio between 6 and 11 when administered through a systemic route in humans [24]. The low aqueous solubility of VRC leads to challenges in formulation and reduced absorption in the gastrointestinal tract. This poor bioavailability necessitates higher doses to achieve therapeutic levels, increasing the risk of adverse effects and drug interactions, particularly CF patients who receive multiple concomitant medications [25]. Overcoming these limitations is crucial for improving the efficacy and safety of VRC in chronic therapy.

Currently, there are commercially available VRC dosage forms for oral and parenteral delivery. However, the oral administration of VRC may induce dose-related side effects (hepatotoxicity, cutaneous malignancies, visual disturbances, and neuro-psychiatric symptoms) [26]. The usefulness of VRC may also be limited by the inability to reach therapeutic levels in the main focus of infection: the lung using the oral route [27]. No inhaling dosage form of VRC is commercially available nowadays. However, inhaled VRC has been used in clinical and investigational settings through compounded nano-formulations or extemporaneous preparations such as VRC suspension [28]. In addition, the pathological CF mucus strongly limits the absorption of drugs and their ability to reach target sites in the lung (bronchi, alveoli).

To date, no VRC formulation has been specifically designed for CF. Existing formulations or drug delivery systems for VRC have been developed with a focus on invasive fungal infections [29,30,31,32,33], which do not involve the pathological mucus environment characteristic of CF patients.

Therefore, more effective drug delivery to mucosal surfaces is needed to achieve optimized antifungal therapy in CF patients. For this purpose, drugs and drug carriers must bypass the adhesive mucus barrier [5].

Nanoparticles (NPs), densely coated with poly(ethylene glycol) (PEG), a muco-inert material, can rapidly diffuse through human mucus secretions [34]. These “mucus-penetrating particles (MPPs)” demonstrated improved mucosal distribution, enhanced pharmacokinetics, and superior treatment efficacy in applications such as CF lung gene therapy [35].

**Figure 1:**
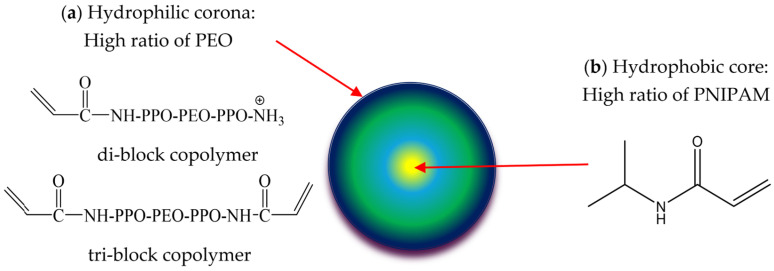
Schematic representation of all NHGs. (**a**) Hydrophilic corona: High ratio of PEO, (**b**) Hydrophobic core: High ratio of PNIPAM.

This study involves the synthesis of series of NHGs based on our established method-ology as a platform for loading VRC as a delivery system for treating broncho-pulmonary fungal infections in CF patients. As the mucus presents a diffusional barrier, the NHGs used here were composed of an outer shell of poly(ethylene oxide) (PEO), which was recently shown to cross lung-generated mucus [34]. An in vitro model of pulmonary pathological mucus that mimics the chemical composition, structural features, and viscoelastic properties of CF mucus [36] allowed us to study their penetration across this diffusional barrier and to assess their activity against the three major molds affecting CF patients worldwide. The biocompatible and biodegradable monodisperse NHG libraries, which range in size from 100 to 400 nm, are disclosed in Figure 1. NHGs are generated through the polymerization of mixtures of N-isopropylacrylamide and di-block (hydrophilic-hydrophobic) and tri-block (hydrophobic-hydrophilic-hydrophobic) copolymer acrylamide macro-monomers made of Jeffamine using recently developed biodegradable cross-linkers [37]. The schematic structure of the NHG is presented in Figure 1, notably a radial distribution of hydrophilic PEO at a high ratio in the outer shell of the NHGs (Figure 1a in blue) and poly N-isopropyl acrylamide (PNIPAM) at a high ratio in the center of the NHGs (Figure 1b in yellow). NHGs possess two significant advantages: mono-dispersity and biocompatibility. Their unique design allows for the fine-tuning and optimization of biological applications by adjusting the particle size within the 100–400 nm range, while maintaining the same particle composition. This tunability offers potential for diverse applications, including targeted drug delivery, imaging, and tissue engineering, where particle size plays a critical role in determining the efficacy and specificity of the intended biological interaction.

## 2. Materials and Methods

Polymerizations were performed in a DAIHAN scientific water bath shaker polymerizer. Jeffamine^®^ ED-2003 (Mn = 1900), acryloyl chloride, polyvinyl-pyrrolidone (PVP_360000_), potassium persulfate (KPS), ethylene glycol, triethanol amine, and Amicon ultrafilter tube (100 kDa cutoff) were purchased from Merck (Jerusalem, Israel); N-isopropylacrylamide (NIPAM, 99.0%) from Acros Organics Co. Ltd. (St. Louis, MI, USA); and Cellulose Ester Spectra/Pore (1000 kDa cutoff) dialysis membrane from Spectrum Laboratories, Inc. (Rancho Dominguez, CA, USA).

Hydrodynamic sizes and size distributions were determined using a Zetasizer 3000 HSA (Malvern Instruments Ltd., Malvern, UK). NP tracking analysis (NTA) was conducted to determine the zeta potential (ζ) and NP number (concentration) using a ZetaView PMX-230 instrument (Particle Metrix GmbH, Inning am Ammersee, Germany). An XTT assay kit (Biological Industries, Beit Haemeq, Israel) was used according to the manufacturer’s instructions. A humidified water-jacketed automatic CO_2_ incubator (US Autoflow, NuAire Inc., Plymouth, MA, USA) was used for cell culture. Dimethyl sulfoxide (DMSO) and VRC were purchased from Merck-Sigma-Aldrich (Buenos Aires, Argentina), and mucin from porcine stomach (type II, powder), sodium alginate, calcium carbonate (CaCO_3_), D-(+)-gluconic acid δ-lactone (GDL), and sodium chloride (NaCl) were used to develop the artificial airway mucus model. All solvents used were of analytical- or high-performance liquid chromatography (HPLC)-grade.

### 2.1. Synthesis of NHGs

The novel NHGs of PNIPAAM-co-PPO-PEO-PPO (Poly(N-isopropylacrylamide)-co-Poly(propylene oxide)-Poly(ethylene oxide)-Poly(propylene oxide)) were obtained via free-radical polymerization using monomeric mixtures of (Acr)1.1Jeffamine1900 and NIPAM, at differing ratios for obtaining different particle sizes, as previously shown [37].

**Figure 2:**
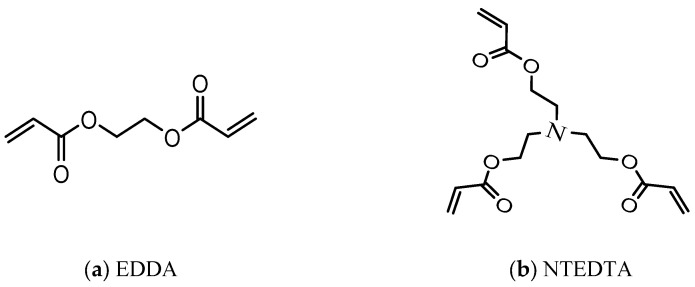
Structure of cross-linkers: (**a**): EDDA; (**b**): NTEDTA.

In addition, for obtaining two different families of biodegradable NHGs, the ester-functionalized cross-linkers (Figure 2), Ethane-1,2-diyl diacrylate (EDDA), and Nitrilot-ris(ethane-2,1-diyl) triacrylate (NTEDTA) were introduced in separate reactions in the presence of potassium persulfate (KPS) as an initiator together with the surfactant poly-vinylpyrrolidone (PVP360000) [37]. These cross-linkers were selected based on their hydrolytically labile ester bonds, which introduce controlled degradability into the polymer backbone under physiological conditions.

E_1–3_/E_4–6_ series present EDDA/NTEDTA cross-linked NHGs, respectively (Table 1). Polymerization was performed at 73 °C in H_2_O (10 mL) with PVP and KPS (5 mg each).

### 2.2. DLS Analysis

Hydrodynamic sizes and size distributions were measured using diluted NHGs (50 µg/3 mL) with a Zetasizer 3000 HSA (Malvern Instruments Ltd., UK) operating with a 4 mW HeNe laser (632.8 nm), a detector positioned at a scattering angle of 90°, and a temperature-controlled cuvette. Three measurements consisting of 10 sub runs were performed for each sample.

### 2.3. Atomic Force Microscopy Characterization

Samples were prepared using the simple spin-coating method. NHGs (1 mg/mL, 50 μL) were coated on a silicon wafer substrate. The coating timer was adjusted for 30 s at 2000 RPM. After spin-coating, samples were thoroughly dried with nitrogen and transferred for AFM imaging (Bruker, GmbH—Karlsruhe, Germany).

### 2.4. Concentration and Zeta Potential (ζ) by NTA

NTA was conducted to determine the ζ and particle number (concentration) of the NPs using the ZetaView instrument described above. Data were collected and analyzed using ZetaView software (version 8.05.16 SP7). Reported particle numbers correspond to the median.

All NHG samples were stored at 4 °C until measurement and were diluted to 1:50,000 in particle-free sterile Milli-Q (MQ) water immediately prior to measurement. The samples were analyzed in light scattering mode using a 488 nm laser. Particles were counted and size-distributed at 25 °C. Instrumental parameters were adjusted as follows: minimum brightness of 30 AU, frame rate of 30 per second (10 per cycle), sensitivity of 85 AU, and shutter of 100 AU. Data for two exposures at 11 measurement positions were collected for each sample. The zeta potential was measured twice at 25 °C with sensitivity of 80 AU and shutter of 150.

### 2.5. Mucus Penetration Assay for NHGs Monitored by NTA

To further investigate the interaction of nano-formulations with mucus, an assay was set to track the movement of single particles through simulated mucus. For this purpose, we used the previously described (Section 2.3) NTA instrument for quantifying the number of NHGs of different sizes that were able to cross a mucus layer prepared as described by Pacheco et al. [36]. To evaluate the NP penetration, an experiment using a dual-chamber model was designed. In the upper compartment, a quantified equal number of NHGs (see Table S1) was placed onto a homogeneous film formed by 100 µL of simulated mucus, supported in a Transwell^®^ insert with a 1 µm microporous membrane. Meanwhile, the lower receiving compartment was filled with 2 mL of PBS. The same setup without the mucus layer was taken as the control. In both cases, the entire setup was incubated at 37 °C for 24 h. At predetermined time intervals (0, 4, and 24 h), 200 µL samples were withdrawn from the lower compartment for analysis. To keep the lower-well volume constant, an appropriate refill of medium (PBS) was performed at each sampling point. For each withdrawn PBS sample, the number of NHGs that managed to pass through the mucus layer was measured with the NTA instrument. Until quantification, samples were preserved at 4 °C, and prior to measurement, dilution in particle-free sterile MQ water was performed, i.e., 1:25 *v*/*v* for samples up to 8 h and 1:2000 *v*/*v* otherwise (up to 24 h).

### 2.6. Loading of VRC

VRC-loaded NHGs (VRC-NHGs) were prepared using the solvent evaporation method [38]. Briefly, VRC (10 mg) was dissolved in chloroform (1 mL), and 200 µL aliquots of the stock solution (10 mg/mL) were dropped into 1 mL of NHGs aqueous solution (10 mg/mL) and mixed overnight at room temperature (RT) to volatilize the chloroform. The obtained NHGs were isolated using an Amicon^®^ ultrafilter tube by centrifugation at 6000 rpm for 20 min and repeated washings with DDW until no signal from the filtrate was detected by UV-visible spectroscopy. The loaded amount of drug was determined using a systematic method. A calibration plot (see Figure S1) was obtained using a set of standard solutions prepared with known concentrations of VRC in acetonitrile. The linear portion of this plot served as a reference to calculate the concentration of drug in NHGs.

The VRC-NHGs (1 mL, 10 mg/mL) were lyophilized for loading calculations. The freeze-dried powder was treated with 5 mL MeCN and filtered to obtain a clear solution that was analyzed by UV spectroscopy. The absorbance was measured at 255 nm, enabling the determination of the loaded amount of drug through the application of the calibration equation derived from the linear portion. This method provides a reliable approach for quantifying encapsulated drug in NHGs [39].

The drug loading efficiency (DLE) and drug loading capacity (DLC) were calculated as:

DLE (%) = (weight of loaded drug/weight of drug used in formulation) × 100(1)

DLC (%) = (weight of encapsulated drug/total weight) × 100(2)

### 2.7. In Vitro Release of VRC

In vitro drug release studies were performed under sink conditions as previously reported [40]. Briefly, VRC-NHGs containing equivalent amounts of VRC were transferred in a suspended dialysis bag to a container for urine analysis containing 100 mL phosphate-buffered saline (PBS) at pH 7.4 and 37 °C with continuous orbital agitation. Samples (1 mL) from the PBS bath were taken at specific intervals (1, 2, 4, 6, 24, 48, 72 h) for quantification of VRC, and an equal amount of fresh PBS was replaced to maintain the volume constant. A solution of free VRC at the same concentration placed in a dialysis bag using the same procedure was used as the control. The amount of VRC released at each interval was analyzed with a fluorimeter (λ_ex_ = 255 nm, λ_em_ = 372 nm). Assays were performed in duplicates, and the results were expressed as the average ± SD.

A number of studies also reported the use of simulated interstitial lung fluid (SILF, pH 7.4) to investigate the release properties of drug formulations due to its close replication of the mucosal fluids in the respiratory system [41,42,43]. A release experiment was also conducted in SILF (pH 7.4) supplemented with 1% *w*/*v* Tween^®^ 80 as previously reported [42]. Dialysis in SILF (pH 7.4) with the addition of 1% *w*/*v* Tween^®^ 80 as external medium was carried out to investigate the in vitro release profiles from the polymeric matrices of the NHGs. SILF was prepared in 1 L of distilled water (pH 7.4): MgCl_2_ (98.5 mg), NaCl (6.032 g), KCl (404 mg), Na_2_HPO_4_ (436 mg), NaHCO_3_ (2.59 g), Na_2_SO_4_ (83 mg), CaCl_2_ (359 mg), sodium acetate (607 mg), and trisodium citrate (115 mg).

### 2.8. Cell Culture

Human lung cancer cell line A549 and embryonic kidney cell line HEK293T were cultured in DMEM medium supplemented with 10% fetal bovine serum (FBS, Sigma-Aldrich, New York, NY, USA), 100 μg/mL streptomycin, and 100 U/mL penicillin at 37 °C in a 5% CO_2_ atmosphere.

### 2.9. Cytotoxicity

The in vitro cytotoxicity of VRC-NHGs was determined in the A549 and HEK293T cell lines using an XTT assay and compared to free VRC. For this purpose, 8 × 10^3^ cells/well were pre-incubated in a 96-well plate for 24 h and incubated for 48 h at 37 °C with 5% CO_2_ and different concentrations of non-loaded NHGs, free VRC, or VRC-NHGs (60 μM VRC). Cells treated with 2% triton were used as the positive control and fresh medium as the negative control. Subsequently, 50 μL of XTT reagent (with initiator) was added, and the cells were incubated for 4 h. The absorbance was read at 570 nm using a TECAN microplate reader.

Cell viability (%) = (I_sample_/I_control_) × 100, where I_sample_ is the absorbance of NHG treated wells and I_control_ is the absorbance of control wells without NHG treatment. Experiments were repeated twice. Assays were carried out according to known procedures [44].

### 2.10. Antifungal Susceptibility Testing

Antifungal susceptibility testing was performed in the mold isolates most frequently recovered from respiratory secretions of CF patients (*A. fumigatus*, *A. flavus*, *A. terreus*, *A. niger*, *A. nidulans*, *S. aurantiacum*, and *E. dermatitidis*) against non-loaded NHGs, VRC-NHGs, VRC suspension, and VRC/DMSO following the clinical laboratory standard institute (CLSI) M38 3rd ed. Document [36]. All clinical fungal isolates included in this study were wild-type with respect to azole antifungals and were collected from spontaneously expectorated sputum samples of CF patients as part of a surveillance study conducted in Argentina [45]. All these isolates are available in the culture collection of the Mycology bank of the Center of Mycology of the School of Medicine (University of Buenos Aires, Argentina). *A. fumigatus* ATCC 204305 (obtained from American Type Culture Collection) was used as the quality control strain for susceptibility testing.

VRC suspension, VRC/DMSO (5 mg/mL), and VRC-loaded NHGs were prepared in sterile saline buffer. Two-fold dilutions of each drug formulation (8 to 0.015 μg/mL) were prepared in 100 μL of RPMI−MOPS in 96-well plates. In total, 5 × 10^4^ CFU/mL were added to each well. The results were recorded 48 h post incubation at 37 °C. The MIC was defined as the lowest drug concentration causing the complete inhibition of visible growth.

Antifungal susceptibilities were interpreted using the available epidemiological cutoff values (ECVs) for VRC and the VRC clinical breakpoint for A. fumigatus published in the CLSI documents M57S-Ed4 and M38M51S-Ed3, respectively, as previously reported by Brito Devoto et al. [45], who characterized the antifungal susceptibility profile of all the clinical strains included in this study.

Aiming at developing an optimized method for the administration of VRC through the mucus barrier, the antifungal activity of VRC-NHGs was evaluated using the CF mucus composition previously described by Pacheco et al. for drug diffusion assays [36]. A 16.3 mg/mL NaCl solution was prepared, and a solution of 21 mg/mL alginate sodium salt was dissolved in it using gentle magnetic agitation. Concurrently, a suspension containing 43.7 mg/mL mucin was meticulously prepared in DDW and left to gently agitate overnight. Subsequently, the alginate and mucin solutions were combined at a 1:4 ratio utilizing interconnected luer-lock syringes, and a suspension of 7 mg/mL CaCO_3_ in the 16.3 mg/mL NaCl solution was intricately blended at 1:5 proportions. A freshly concocted 70 mg/mL GDL solution in 16.3 mg/mL NaCl was merged with the previously created suspension (comprising alginate, mucin, and CaCO_3_) at 1:6 proportions, always under aseptic conditions. Lastly, 100 μL of the prepared mucus was accurately pipetted onto the 96-well surface, previously inoculated with fungal conidia. The different VRC formulations (VRC-NHGs, VRC suspension, and VRC/DMSO) were added onto the surface of the mucus layer, finalizing the experimental procedure. This antifungal susceptibility procedure was performed according to the abovementioned CLSI document against *A. fumigatus* as the most common filamentous fungus isolated from the airways of CF patients [46].

## 3. Results and Discussion

### 3.1. Physico-Chemical Characterization of NHGs and VRC-NHGs

Chronic pulmonary infections caused by opportunistic pathogens affect more than 90% of CF patients. In addition to bacterial colonization, these patients are predisposed to fungal colonization due to the capacity of environmental fungi to colonize the lower respiratory tract and to the frequent cycling of antibiotics required to control the disease.

One of the main goals of the present study was the synthesis of a series of NHGs based on our established methodology as a platform for loading the antifungal drug VRC as a delivery system for an optimized antifungal therapy of broncho-pulmonary infections caused by molds in CF patients. As the mucus presents a diffusional barrier to the antifungal treatment, the NHGs used in this study were composed of a shell of PEG, recently shown to be capable of crossing lung-generated mucus [34].

With this in mind, we developed novel polymeric monodispersed NHGs with a large range of sizes starting from 100 nm up to 400 nm, which according to previous results exhibited high biocompatibility both in vitro and in vivo [37,39,47,48]. This non-toxic profile may support its potential use for treating fungal infections especially in CF patients.

In the present study, monomeric mixtures in water at RT spontaneously formed star/flower-like self-assemblies with uncontrollable poly-dispersed sizes around 100–800 nm. Upon heating at 75 °C, NIPAM and the hydrophobic elements of the macro-monomers (PPO) collapse together into well-defined self-assemblies probably with a high concentration of the hydrophobic elements in the core (NIPAM and PPO chains) and a high concentration of the hydrophilic elements of the macro-monomeric chains (PEO and NH2 groups) at the outer shell. These self-assemblies can be polymerized, leading to particles with sizes that correlate with those of the intermediate self-assemblies. The size of the assemblies can be tuned/calibrated by combining different ratios of the starting monomeric mixtures at high temperature, as previously reported [37,39,47,48]. Larger amounts of (Acr)1.1Jeffamine1900 correspond to smaller NHG diameters due to increased hydrophobic interactions during self-assembly.

Dh represents the particle size of pristine and VRC-NHGs as measured by DLS at 25 °C, and the polydispersity index (PDI) represents the relative variance in the particle size distribution [49,50]. Each value is the average of three measurements by DLS. The results shown in Table 2 indicate that both EDDA cross-linked (series E_1–3_) and NTEDTA cross-linked NHGs (series E_4–6_) are highly monodisperse and thermo-responsive [37].

The D_H_ of the VRC-NHGs is a key parameter for their potential clinical use as nanocarriers in CF patients. Carriers in the nano scale have a greater potential to penetrate the airway mucus of CF patients than microparticles, avoiding steric interactions [51]. Thus, VRC-NHGs can overcome the mucus barrier for a better treatment outcome. The data summarized in Table 2 show that all VRC-NHGs maintain their size upon encapsulation. For instance, the E1 batch of NHGs and VRC-NHGs span 410 and 424 nm, respectively. In both cases, there is a unimodal narrow size distribution according to the PDI (0.17/0.12 for NHGs/VRC-NHGs).

The zeta potential measures the electrostatic charge on the surface of particles in a colloidal dispersion (at the shear plane of the particle) [49,50]. The potentials of NHGs and VRC-NHGs were examined by NTA at 25 °C, pH 7.4. All ζ measured at neutral pH were clearly negative (Table 2). This negativity is attributed to the presence of sulfate groups generated at the starting polymeric chains by KPS initiator that are accommodated at the shear plane of the NHGs. A negative zeta potential offers several benefits for NPs. It enhances stability by repelling charges, preventing aggregation, and ensuring a more uniform dispersion, crucial for colloidal stability [52,53]. In biomedical applications, NPs with higher negative potential typically show improved bioavailability. Cellular uptake and intracellular trafficking can also be influenced, providing better control over drug release kinetics and targeting specificity, which is particularly advantageous for drug delivery systems [52]. Moreover, the reduced interaction with cells and tissues due to a high negative ζ can potentially lower the toxicity of NPs, enhancing their biocompatibility, particularly in biomedical imaging, diagnostics, and therapeutics [53].

**Figure 3:**
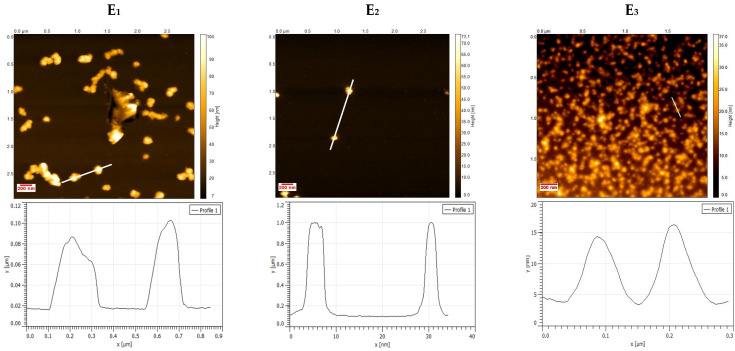

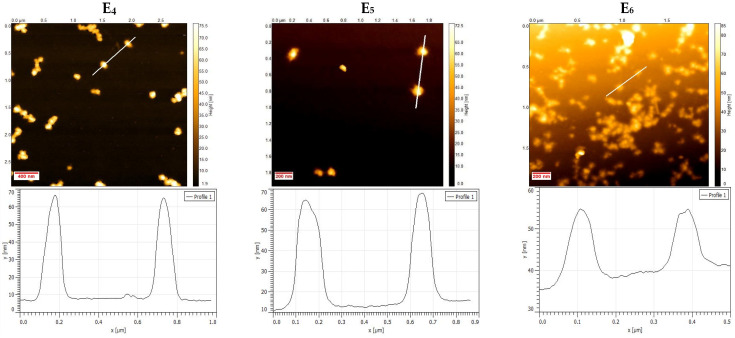
AFM topography images of E_1–3_ (**top**) and E_4–6_ (**bottom**) NHGs.

AFM analysis characterization of E_1–3_ and E_4–6_ NHGs (Figure 3) showed spherical and monodispersed particles. Line profiles from the marked region in the AFM image indicate smaller sizes on a Si wafer compared to DLS (Table 2), as we expected due to the NHGs dryness during AFM measurement.

### 3.2. Mucus Penetration Assay of NHGs Monitored by NTA

**Figure 4:**
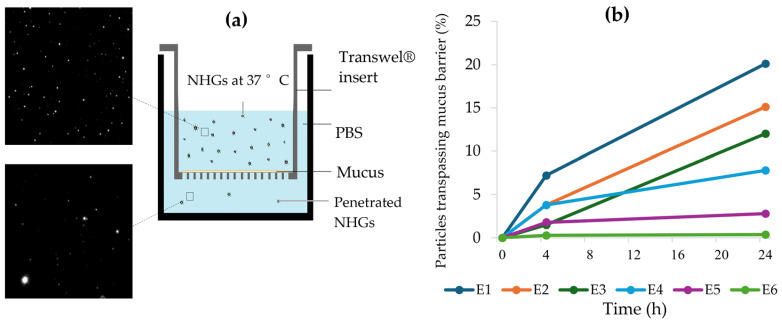
(**a**) Illustration of double-chamber setup and NTA images. (**b**) Percentage of NHG particles trespassing mucus over time.

The goal of this experiment was to evaluate NHG diffusion through artificial mucus using a dual-chamber setup (Figure 4a). PBS samples taken from the lower compartment at specific intervals (0, 4, and 24 h) were measured by NTA to quantify the particles reaching that compartment after crossing the mucus layer.

The entire setup was incubated at 37 °C for 24 h. As our NHGs are based on thermo-responsive poly(N-isopropylacrylamide) (PNIPAM), exposure to temperatures above the low critical solution temperature (LCST) value of 37 °C induces a reversible phase transition in the polymer chains—from a swollen coil state to a collapsed globule. This thermally driven transition can be utilized in an on–off delivery system [54,55,56]. In other words, the NHGs decrease in size when the temperature exceeds the LCST. Consequently, under the experimental conditions, all NHGs were smaller than the mesh size of the mucus layer [37].

Percentages (see Table 3 and Table S2) were calculated relative to the total number of particles on the receptor media of the same setup without mucus (Figure 4b). The results are shown in Table 3 and Figure 4b as percentages of an equally added number of particles crossing the mucus over time.

Series E_1–3_ shows, in general, better penetration than series E_4–6_. As for the size, larger NPs display more penetration for both series. We speculate that smaller particles seem to exhibit more interaction with the mucus film interstices and therefore spend more time within this layer compared to larger ones, in contrast to previous reports [57,58].

Our findings support recent results [59], which showed that larger PEGylated nanoparticles (200 and 500 nm) penetrate mucus more effectively than smaller counterparts. This observation suggests two possible explanations: (1) the mucus mesh spacing may be significantly larger and more heterogeneous than previously believed, and/or (2) mucus may exhibit an “exclusion” mechanism similar to exclusion chromatography. In this model, particles navigate through two distinct pathways—one consisting of tightly condensed regions with small mesh pores that trap smaller particles, and another comprising larger mesh channels that allow rapid transit of larger particles. Regardless of the mechanism, PEG-coated large nanoparticles appear to diffuse through low-viscosity channels without substantially disrupting mucus structure.

In addition to enhanced transport, larger nanoparticles offer other benefits, including higher drug-loading capacity, increased stability during formulation and storage, and more sustained drug release profiles. However, while larger sizes confer these advantages, excessively large particles may suffer from limited diffusion and decreased cellular uptake. Therefore, optimizing nanoparticle size is essential to maximize therapeutic efficacy across various mucosal tissues [59].

### 3.3. VRC Loading into NHGs

In all loading experiments, the applied amount of VRC was 2 mg in 10 mg of NHGs. The DLE and DLC were calculated according to these parameters. The results in Table 4 indicate that larger NHGs have a better drug loading capacity that is directly proportional to their increased hydrophobicity due to their higher content of PNIPAM in their structure as measured by NMR in a previous report [37]. Both series display a similar loading capacity.

Overall, both NP hydrophobicity and size, as well as the hydrophobic nature of the drug, contribute to the drug loading capacity [60].

### 3.4. In Vitro Drug Release Study of VRC-NHGs

**Figure 5:**
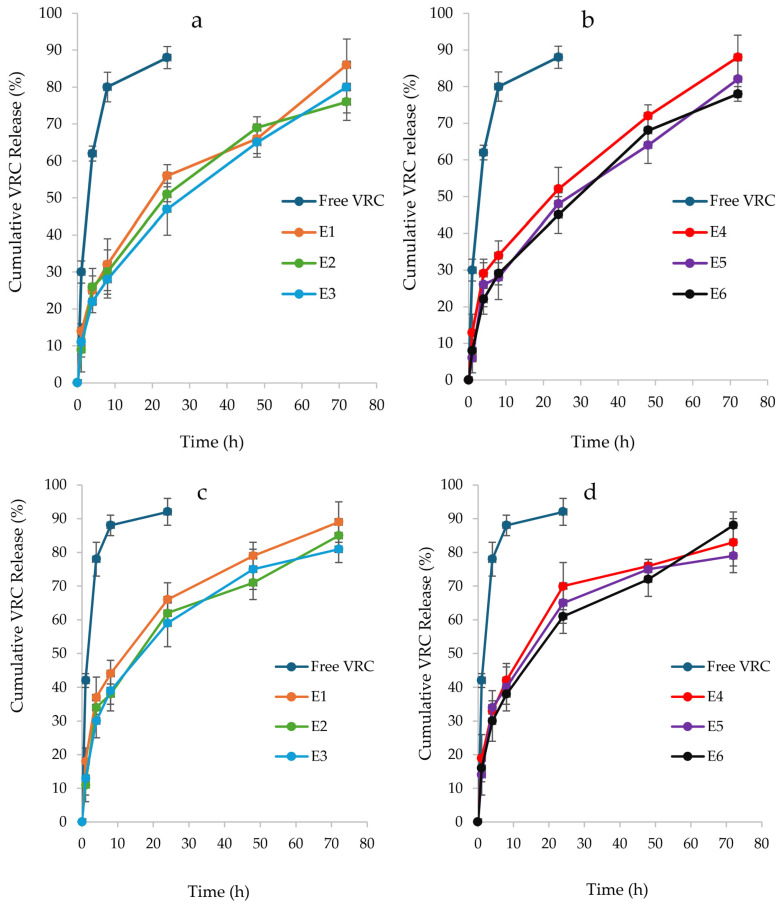
In vitro release profiles of VRC from NHGs: (**a**) (E_1–3_) and (**b**) (E_4–6_) in PBS; (**c**) (E_1–3_) and (**d**) (E_4–6_) in SILF environment. Data represents the mean ± SD, (*n* = 2).

To investigate the drug release from the NHGs, the fluorescence of released VRC was measured at predetermined time intervals (λ_ex_ = 255 nm, λ_em_ = 372 nm). VRC-NHGs and free VRC as the control were dialyzed in PBS (50 mL), as largely studied by others under sink conditions [40,61]. VRC-NHGs from both series (Figure 5a,b) showed similar release profiles with approximately 50% of encapsulated VRC released within 24 h and around 80% released after 72 h. In contrast, the free VRC control demonstrated a more rapid release, exceeding 80% after just 8 h, emphasizing the role of NHGs in facilitating a controlled release mechanism.

A release experiment was also conducted in SILF (pH 7.4), supplemented with 1% *w*/*v* Tween^®^ 80 as previously reported (Figure 5c,d) [42]. This external medium was utilized to explore the in vitro release profiles of VRC. Here, we dialyzed VRC-NHGs and free VRC as the control in SILF (50 mL), as largely studied by others under sink conditions [41,42,43].

In the SILF environment, both series of VRC-NHGs exhibited similar release profiles (Figure 5c,d) with over 60% of encapsulated VRC released within 24 h and ~85% after 72 h. The free VRC control exhibited a release of 85% after 8 h. This further highlights the role of NHGs in enabling a sustained release of VRC.

The slightly faster release of VRC observed in the SILF environment compared to the PBS environment can be attributed to its ionic composition, which closely mimics lung interstitial conditions, enhancing drug solubility and diffusion.

VRC exhibits very low solubility in phosphate-buffered saline (PBS), which can limit its release from nanohydrogel formulations in such environments. In PBS, the release of VRC from nanohydrogels is primarily governed by the drug’s dissolution rate, which is constrained due to its poor solubility in aqueous media. This limitation affects the diffusion of VRC from the nanohydrogel matrix into the surrounding medium.

In contrast, when evaluating VRC release in SILF, the addition of 1% *w*/*v* Tween^®^ 80 to the release medium was employed. Tween^®^ 80, a non-ionic surfactant, enhances the solubility of hydrophobic drugs like VRC by forming micelles that encapsulate the drug molecules, thereby increasing their apparent solubility in aqueous environments. This micellar solubilization facilitates a more efficient release of VRC from the nanohydrogel into the SILF medium.

Moreover, the ionic composition of the release medium can influence the swelling behavior of hydrophilic polymers within the nanohydrogel, potentially affecting drug release dynamics. However, the primary role of Tween^®^ 80 in this context is to improve the solubility and release profile of VRC by mitigating its inherent hydrophobicity, rather than directly altering the ionic characteristics of the medium.

In summary, the incorporation of Tween^®^ 80 into the SILF release medium serves to enhance the solubility and subsequent release of VRC from nanohydrogel formulations, addressing the solubility challenges posed by the drug’s hydrophobic nature.

### 3.5. Cytotoxicity Studies of NHGs and VRC-NHGs

**Figure 6:**
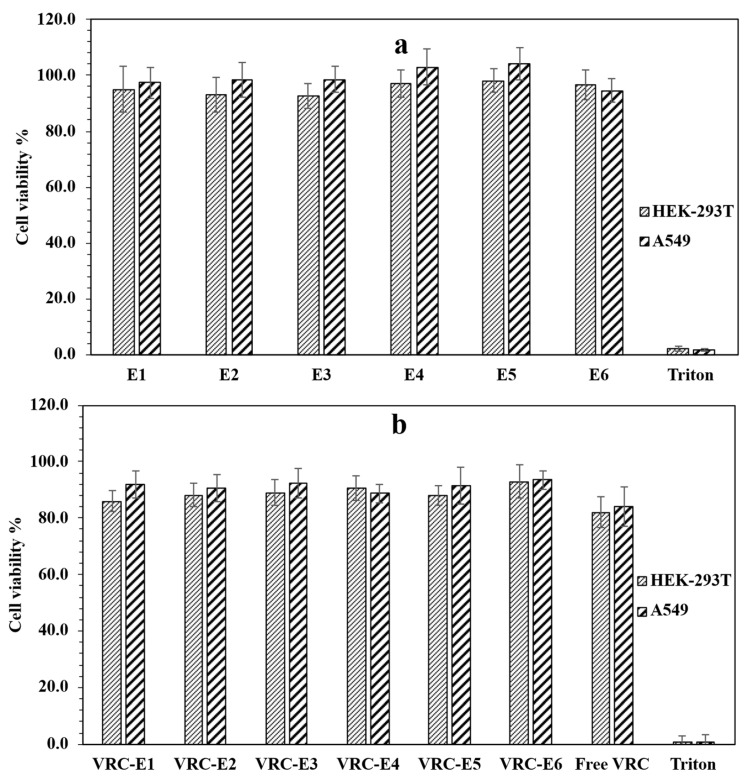
In vitro cytotoxicity of (**a**) NHGs and (**b**) VRC-NHGs (20 µg/mL) at 48 h incubation in HEK293T and A549 cell lines. Each column represents the mean ± SD (n = 3).

Ensuring the safety and compatibility of the formulation with lung cells is essential for developing a pulmonary drug delivery system, as inhalation of toxic substances may deplete lung surfactant and trigger phagocytic cell activation [62]. Both NHGs and VRC-NHGs were tested for cell toxicity using XTT viability assay and were compared to the relevant controls on HEK-293T and A549 cell lines. The results demonstrate that NHGs (Figure 6a) are devoid of toxicity at concentrations of 2 mg/mL on A549 and HEK293T cell lines after incubating for 48 h. Both cell lines show beyond 90% viability. This concentration is by far higher than those reported for most of the NPs reported as having low toxicity [28]. VRC-NHGs are non-toxic with 20 µg/mL of VRC on A549 and HEK-293T after incubating for 48 h (Figure 6b).

Both cell lines show beyond 80% viability. The viability of the control group (free VRC: 20 µg/mL) was 80–84%.

These results show that VRC-NHGs could be considered a “safe nano-formulation” where cell viability is not compromised. Cell viability above 70% is considered safe in vitro assays according to the ISO 10993-5 guideline [63].

### 3.6. Antifungal Effect of VRC-NHGs

The antifungal studies showed that only 400 nm VRC-NHGs (see E_1_ and E_4_ in Table 5) maintained high activity, comparable to free VRC in DMSO and up to eight-fold higher than the aqueous suspension. For instance, 400 nm VRC-NHGs exhibited lower MIC values against *A. fumigatus* (four-fold), *A. flavus* (four-fold), *A. terreus* (four-fold), *A. niger* (four-fold), *A. nidulans* (four-fold), *S. aurantiacum* (four-fold), and *E. dermatitidis* (eight-fold) than those MIC values observed for the drug aqueous suspension (Table 5). These data might be related to the capacity of the NHGs to increase VRC aqueous solubility and sustain its release compared to the drug suspension where VRC is homogenously dispersed (but not solubilized) in aqueous medium. The non-loaded NHGs did not display any antifungal activity against any of the studied strains. Activity decreases proportionally with size, resulting in a MIC similar to that of VRC suspension (see E_2_, E_3_, E_5_, and E_6_ in Table 5).

By using an in vitro model of pulmonary pathological mucus that mimics the chemical composition, structural features, and viscoelastic properties of CF mucus [36], we studied their penetration across this diffusional barrier and assessed the antimicrobial capacity of the developed NHGs against A. fumigatus, which is the most frequent fungal agent affecting CF patients worldwide. The results indicate that the introduction of a mucus barrier for A. fumigatus (denoted by a star) did not significantly alter the antifungal effect of all VRC-NHGs, which remained comparable to that observed under conditions without the mucus barrier. These results are in line with chitosan-based polymeric micelles for the pulmonary delivery of itraconazole and with Soluplus-based polymeric micelles for the pulmonary delivery of VRC, where the micellar system and a drug solution in DMSO were compared [64,65].

Furthermore, these data suggest that the encapsulation of VRC within the 400 nm NHGs have an improved antimicrobial potential to the aqueous suspension itself, considering that nowadays only an aqueous VRC suspension and a dug parenteral solution represent the commercially available dosage forms.

Regarding the mucus penetrating assay, the results suggest that the 400 nm NHGs (E_1_ and E_4_) can penetrate through the mucus better, as shown also for the non-loaded NHGs in Figure 4, serving as a sustained release depot, improving the antifungal efficacy of VRC against the tested major molds. It should be further explored whether these mucus-penetrating NHGs, containing different hydrophobic therapeutics effectively bypass the mucus barrier, allowing better drug distribution and absorption at mucosal surfaces.

The ability of these novel 400 nm NHGs to bypass the mucosal barrier offers a significant advantage for the treatment of CF, where highly viscoelastic mucus presents a major diffusional barrier relevant to drug delivery challenges in CF.

These results are promising, as our VRC nano-formulation demonstrated improved in vitro antifungal activity, overcoming a physical barrier like the artificial CF mucus. The data denote the potential of our 400 nm NHG system for enhanced clinical management of CF patients employing a VRC respirable nanocarrier. In particular, the MIC results observed for the VRC-NHG formulation were both lower than the VRC clinical breakpoint for A. fumigatus (1 μg/mL), regardless of the presence of the mucus layer, and lower than the VRC ECV described for the other fungal species (1 µg/mL for *A. flavus* and *A. terreus*, and 2 µg/mL for *A. niger* and *A. nidulans*). Currently, there are no established ECVs for VRC specifically for *S. aurantiacum* and *E. dermatitidis*. In a study involving clinical isolates of various Scedosporium [66] species, including *S. aurantiacum*, VRC demonstrated significant activity, with an MIC of ≤2 μg/mL for most isolates. Another study evaluating 51 E. dermatitidis strains isolated from CF patients found that the MIC range for VRC was 0.03 to 0.5 µg/mL, suggesting good in vitro activity for the drug [67].

Then, VRC-loaded NHGs could be effective in reducing pulmonary fungal load and improving clinical outcome in CF patients.

## 4. Conclusions

Ester-functionalized cross-linked NHGs with tunable sizes (100–400 nm), and devoid of toxicity, were prepared by free radical polymerization. The present study demonstrates that the newly developed NHGs are effective for controlled drug delivery, as evidenced by the in vitro controlled release of VRC-NHGs under different sink conditions.

The 400 nm VRC-NHGs (E_1_ and E_4_) exhibit high antifungal activity, comparable to free VRC in DMSO and superior to an aqueous VRC suspension. Notably, even in the presence of a mucus barrier, the antifungal effect remained consistent. This research showcases the significant advancements in the field of biodegradable NHGs and their potential for diverse biological applications, such as the therapy of lung disorders caused by fungi in CF patients. These findings are at the basis of ongoing in vivo investigations aimed at exploring the applications and advantages of these promising NHGs.

## Figures and Tables

**Table 1:** Synthesis of E_1–3_ and E_4–6_ NHG series (mg) [37].

#	(Acr)_1.1_Jeffamine_1900_	NIPAM	EDDA	NTEDTA
**E_1_**	130	120	15	-
**E_2_**	260	120	15	-
**E_3_**	320	120	15	-
**E_4_**	200	188	-	19
**E_5_**	300	188	-	19
**E_6_**	400	188	-	19

**Table 2:** Physical properties of NHGs and VRC-NHG series at 25 °C. D_h_ [nm] results represent the mean ± SD, (n = 3).

#	NHGs				VRC-NHGs			
	D_h_ [nm]	PDI	ζ [mV]	NPs/mL	D_h_ [nm]	PDI	ζ [mV]	NPs/mL
**E_1_**	410 ± 4	0.17	−27.9	5.7 × 10^12^	424 ± 2	0.12	−21.7	1.4 × 10^12^
**E_2_**	242 ± 8	0.28	−28.8	7.4 × 10^12^	251 ± 6	0.19	−9.2	4.8 × 10^12^
**E_3_**	136 ± 5	0.17	−23.3	3.7 × 10^12^	145 ± 5	0.22	−27.5	5.2 × 10^12^
**E_4_**	392 ± 2	0.08	−27.1	1.3 × 10^12^	407 ± 4	0.14	−26.7	1.6 × 10^12^
**E_5_**	198 ± 6	0.15	−27.3	2.4 × 10^12^	220 ± 5	0.23	−23.5	6.7 × 10^12^
**E_6_**	121 ± 7	0.24	−11.9	6.4 × 10^12^	134 ± 3	0.19	−20.0	5.6 × 10^12^

**Table 3:** Percentage of NHGs crossing the mucus barrier over time.

% Particles Trespassing Mucus Barrier						
Time [h]	E_1_	E_2_	E_3_	E_4_	E_5_	E_6_
**4**	7.2	3.8	1.5	3.8	1.8	0.3
**24**	20.1	15.1	12	7.8	2.8	0.4

**Table 4:** Drug loading efficiency and capacity of NHG series. Data represent the mean ± SD, (n = 3).

#	Loaded VRC [µg]	DLE [%]	* DLC [%]
**E_1_**	325 ± 8	16.2	3.2
**E_2_**	276 ± 9	13.8	2.8
**E_3_**	208 ± 5	10.4	2.1
**E_4_**	312 ± 10	15.6	3.1
**E_5_**	245 ± 7	12.2	2.4
**E_6_**	198 ± 6	9.9	2.0

* In all cases, 2 mg of drug was applied into 10 mg of NHGs. DLE/DLC was calculated accordingly.

**Table 5:** MIC (µg/mL) values of VRC-NHGs (no mucus layer). Each value is the mean of two measurements.

Fungal Species	VRC-DMSO	VRC Susp.	VRC-E_1_	VRC-E_2_	VRC-E_3_	VRC-E_4_	VRC-E_5_	VRC-E_6_
*A. fumigatus*	0.25	2	0.5	1	1	0.5	1	2
* A. fumigatus	0.25	2	0.5	1	1	0.5	1	2
*A. terreus*	1	2	0.5	1	2	0.5	1	2
*A. flavus*	0.5	2	0.5	1	2	0.5	1	2
*A. niger*	0.5	2	0.5	1	2	0.5	1	2
*A. nidulans*	0.5	2	0.5	1	1	0.25	1	2
*E. dermatitidis*	0.5	2	0.25	1	1	0.25	1	2
*S. auarantiacum*	0.5	2	0.5	1	2	0.5	1	1

* The same activity was found in the presence of a mucus layer (the second row).

## Data Availability

The data presented in this study are available on request from the corresponding author due to patent registration.

## Supplementary Materials

The following supporting information can be downloaded at https://www.mdpi.com/article/10.3390/pharmaceutics17060725/s1. Figure S1: Calibration curve of VRC/MeCN for quantification of loading VRC in NHGs; Table S1: Initial number of NHGs for mucus penetration assay; Table S2: Percentage of NHGs crossing the mucus barrier over time.
