# Supplementary material for: Voriconazole-Loaded Nanohydrogels Towards Optimized Antifungal Therapy for Cystic Fibrosis Patients
Source: Pharmaceutics. 2025 May 30;17(6):725. doi: 10.3390/pharmaceutics17060725 (PMC12196158; doi:10.3390/pharmaceutics17060725)
Supplement: Supplementary file 1 [file pharmaceutics-17-00725-s001.zip › pharmaceutics-3632573-supplementary.pdf]

pharmaceutics

Supplementary Information: Preparation and characterization of voriconazole-loaded nanohydrogels towards an optimized antifungal therapy for cystic fibrosis patients

Shaul D. Cemal [1†], María F. Ladetto [2,3†], Katherine Hermida Alava [2], Gila Kazimirsky, Marcela Cucher [2], Romina J. Glisoni [4], María L. Cuestas [2,*] and Gerardo Byk [1,*]

[1]Laboratory of Nano-Biotechnology. Dept. of Chemistry Bar-Ilan University.
[2]Universidad de Buenos Aires. CONICET. Instituto de Investigaciones en Microbiología y Parasitología Médica (IMPaM). Buenos Aires, Argentina.
[3]Centro de Investigación y Desarrollo en Fermentaciones Industriales (CINDEFI), Laboratorio de Nanobiomateriales, Departamento de Química, Facultad de Ciencias Exactas, Universidad Nacional de La Plata (UNLP) -CONICET (CCT La Plata), Calle 47 y 115, (B1900AJI) La Plata, Buenos Aires, Argentina.
[4]Universidad de Buenos Aires. CONICET. Instituto de Nanobiotecnología (NANOBIOTEC). Buenos Aires, Argentina.

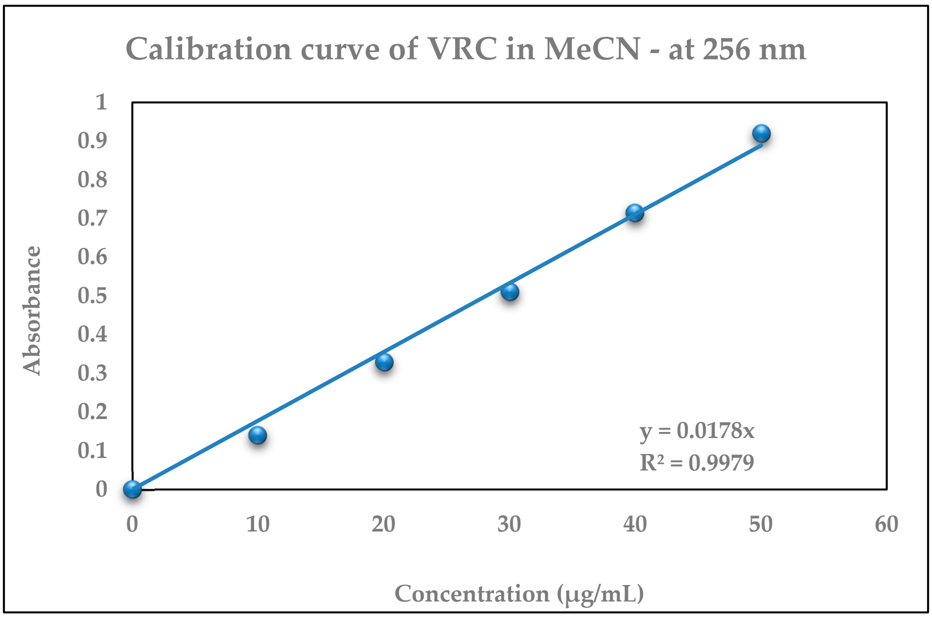

Figure S1. Calibration curve of VRC/MeCN for quantification of loading VRC in NHGs.

VRC/MeCN calibration curve were created by preparing a set of standard solutions with known concentrations of the analytes. The linear portion of this plot served as a reference to calculate the concentration of drug in NHGs by correlating their response to concentration.

Table S1. Initial number of NHGs for mucus penetration assay.

#	Sample t = 0
E_1	1.4(±0.3) E+12
E_2	1.2(±0.3) E+12
E_3	1.1(±0.3) E+12
E_4	1.6(±0.3) E+12
E_5	1.1(±0.3) E+12
E_6	1.1(±0.3) E+12

Table S2. Percentage of NHGs crossing the mucus barrier over time.

Time [h]	% Particles trespassing mucus barrier																	
	E_1			E_2			E_3			E_4			E_5			E_6		
	[Nº $_{NP}$/mL]		%	[NºNP/mL]		%	[NºNP/mL]		%	[NºNP/mL]		%	[NºNP/mL]		%	[NºNP/mL]		%
	without mucus	with mucus		without mucus	with mu-cus		without mucus	with mu-cus		without mucus	with mu-cus		without mucus	with mu-cus		without mucus	with mu-cus	
**4**	2.7×10^{11}	2.0×10^{10}	7.2	3.7×10^{11}	1.4×10^{10}	3.8	3.5×10^{11}	5.3×10^{9}	1.5	1.2×10^{11}	4.6×10^{9}	3.8	3.3×10^{11}	5.9×10^{9}	1.8	2.5×10^{11}	7.5×10^{8}	0.3
**24**	2.8×10^{11}	5.6×10^{10}	20.1	3.7×10^{11}	5.6×10^{10}	15.1	3.6×10^{11}	4.2×10^{10}	12	1.2×10^{11}	9.4×10^{9}	7.8	3.3×10^{11}	9.3×10^{9}	2.8	2.7×10^{11}	1.1×10^{9}	0.4
